# Antibiotic resistance associated with the COVID-19 pandemic: a systematic review and meta-analysis

**DOI:** 10.1016/j.cmi.2022.12.006

**Published:** 2023-03

**Authors:** Bradley J. Langford, Jean-Paul R. Soucy, Valerie Leung, Miranda So, Angela T.H. Kwan, Jacob S. Portnoff, Silvia Bertagnolio, Sumit Raybardhan, Derek R. MacFadden, Nick Daneman

**Affiliations:** 1) Public Health Ontario, Toronto, Ontario, Canada; 2) Dalla Lana School of Public Health, University of Toronto, Toronto, Ontario, Canada; 3) Toronto East Health Network, Toronto, Ontario, Canada; 4) Leslie Dan Faculty of Pharmacy, University of Toronto, Toronto, Ontario, Canada; 5) Department of Medicine, University of Ottawa, Ottawa, Ontario, Canada; 6) Faculty of Medicine, University of Queensland, Brisbane, Queensland, Australia; 7) Department of Surveillance, Prevention and Control, Division of Antimicrobial Resistance, World Health Organization, Geneva, Switzerland; 8) North York General Hospital, Toronto, Ontario, Canada; 9) The Ottawa Hospital, Ottawa, Ontario, Canada; 10) Institute of Health Policy, Management and Evaluation, University of Toronto, Toronto, Ontario, Canada; 11) Sunnybrook Health Sciences Centre, Toronto, Ontario, Canada

**Keywords:** Antibiotic resistance, Antimicrobial resistance, Antimicrobial stewardship, COVID-19, Infection, Prevention and control

## Abstract

**Background:**

COVID-19 and antimicrobial resistance (AMR) are two intersecting global public health crises.

**Objective:**

We aimed to describe the impact of the COVID-19 pandemic on AMR across health care settings.

**Data source:**

A search was conducted in December 2021 in WHO COVID-19 Research Database with forward citation searching up to June 2022.

**Study eligibility:**

Studies evaluating the impact of COVID-19 on AMR in any population were included and influencing factors were extracted. Reporting of enhanced infection prevention and control and/or antimicrobial stewardship programs was noted.

**Methods:**

Pooling was done separately for Gram-negative and Gram-positive organisms. Random-effects meta-analysis was performed.

**Results:**

Of 6036 studies screened, 28 were included and 23 provided sufficient data for meta-analysis. The majority of studies focused on hospital settings (*n* = 25, 89%). The COVID-19 pandemic was not associated with a change in the incidence density (incidence rate ratio 0.99, 95% CI: 0.67–1.47) or proportion (risk ratio 0.91, 95% CI: 0.55–1.49) of methicillin-resistant *Staphylococcus aureus* or vancomycin-resistant enterococci cases. A non-statistically significant increase was noted for resistant Gram-negative organisms (i.e. extended-spectrum beta-lactamase, carbapenem-resistant Enterobacterales, carbapenem or multi-drug resistant or carbapenem-resistant *Pseudomonas aeruginosa* or *Acinetobacter baumannii*, incidence rate ratio 1.64, 95% CI: 0.92–2.92; risk ratio 1.08, 95% CI: 0.91–1.29). The absence of reported enhanced infection prevention and control and/or antimicrobial stewardship programs initiatives was associated with an increase in gram-negative AMR (risk ratio 1.11, 95% CI: 1.03–1.20). However, a test for subgroup differences showed no statistically significant difference between the presence and absence of these initiatives (p 0.40).

**Conclusion:**

The COVID-19 pandemic may have hastened the emergence and transmission of AMR, particularly for Gram-negative organisms in hospital settings. But there is considerable heterogeneity in both the AMR metrics used and the rate of resistance reported across studies. These findings reinforce the need for strengthened infection prevention, antimicrobial stewardship, and AMR surveillance in the context of the COVID-19 pandemic.

## Graphical abstract

**Figure:**
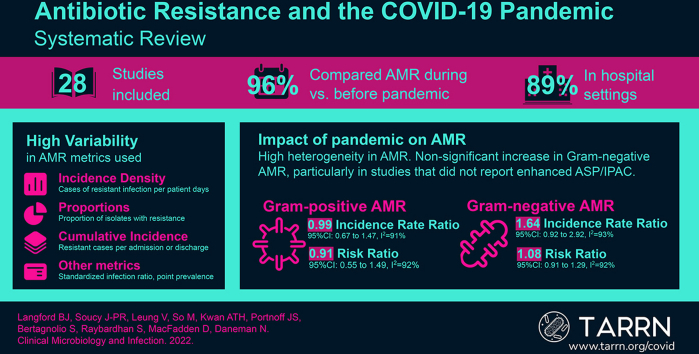

## Background

High antibiotic use among patients with COVID-19 threatens to contribute to the antimicrobial resistance (AMR) crisis. Although antibiotics do not treat COVID-19, they are commonly used because of initial diagnostic uncertainty in patients presenting with respiratory illness and of concern for bacterial co-infection or secondary infection in those with confirmed COVID-19. In previous rapid reviews, we found high antibiotic prescribing (approximately 75%) to patients with COVID-19 despite the relatively low proportion of patients with bacterial infection, particularly among patients outside of the intensive care unit setting (<10%) [[1], [2], [3]].

Our most recent systematic review identified patients with COVID-19 as a potentially important reservoir for AMR. More than 60% of patients with COVID-19 who had a bacterial infection carried a highly resistant organism [4]. Due to person-to-person transmission of organisms, particularly in health care settings, this presents a threat to the broader population beyond those with COVID-19.

Although substantial inappropriate antibiotic prescribing has occurred in patients with COVID-19, antibiotic use for other infectious syndromes has declined early in the pandemic, particularly in community settings [5,6]. This could potentially be due to the attenuation of transmission of other viral and bacterial pathogens due to public health measures to contain COVID-19, including physical distancing and masking. Enhanced infection prevention and control activities in health care settings could further mitigate the impact on AMR [7]. Given potentially opposing effects, it is unclear how the selection of AMR in bacteria has occurred across populations during the pandemic. Emerging data from the United States Centers for Disease Control and Prevention suggests the pandemic has resulted in rising rates of AMR, including carbapenem-resistant *Acinetobacter* and extended-spectrum beta-lactamase-producing Enterobacterales [8].

Although we have reported that antimicrobial resistance is high in individual patients with COVID-19 and bacterial infection, the ecological impact of the pandemic on AMR at the population level is not yet well-described. In this analysis, we present the findings of a systematic review and meta-analysis describing the impact of the COVID-19 pandemic on AMR across health care settings.

## Methodology

### Searches

We performed a comprehensive search of the WHO COVID-19 Research Database for published literature in any language from 1 January 2019 to 1 December 2021 (PROSPERO registration: CRD42022325831). The WHO COVID-19 Research Database is a comprehensive multilingual source of COVID-19 literature updated weekly that includes citations from Medline, Scopus, CINAHL, ProQuest Central, Embase, and Global Index Medicus [9]. The search strategy was structured to include co-infection or secondary infection terms and bacterial infection terms which were applied to the COVID-19 literature in the database. The full search strategy is available in the supplement. Forward citation searching was performed in Google Scholar to capture more recent publications up to June 2022 [10].

### Study eligibility

All studies in inpatient and outpatient settings were eligible for inclusion. The following inclusion and exclusion criteria were applied.

### Inclusion criteria

1. Study provides data on AMR before (before January 2020, or as identified by authors) vs. during the COVID-19 pandemic (January 2020 or later, or as identified by authors) in a specific health care setting.

2. AMR is reported as (a) incidence density rate (e.g. rate per 1000 patient days or per patient population), and/or (b) effect measure (e.g. risk, odds, rate ratio) of AMR, and/or (c) prevalence of antimicrobial resistant organisms (e.g. methicillin-resistant *Staphylococcus aureus* (MRSA) out of all *Staphylococcus aureus*).

AMR includes any of the following pathogens and resistance phenotypes, as defined by study authors: MRSA, vancomycin-resistant enterococci (VRE), carbapenem or multi-drug resistant (MDR) *Pseudomonas aeruginosa.*, carbapenem or MDR *Acinetobacter baumannii.*, extended-spectrum beta-lactamase (ESBL)-producing (or third-generation cephalosporin-resistant) Enterobacterales, carbapenem-resistant Enterobacterales (CRE).

### Exclusion criteria

1. Reviews, editorials, case studies, case series, letters, pre-print publications, dissertations, and poster presentations.
2. Studies including <100 patients.

### Population

Individuals receiving care in any health care setting and any age group.

### Main outcomes

The main outcome is the incidence of AMR in the population associated with the COVID-19 pandemic, either expressed as an incidence density rate (antibiotic-resistant infections per 1000 patient days) or proportion (e.g. proportion of *S. aureus* that was MRSA, the proportion of patient admissions with resistant infection).

### Data screening and extraction

Records were managed using Covidence bibliographic software. All titles and abstracts were screened by a single author (in our previous review, there was substantial reviewer agreement, kappa: 0.66). The full-text screening was performed by at least a single author (in the previous review, we determined kappa to be substantial at 0.62–0.68). A single review author extracted study characteristics and data according to a pre-defined list of study elements, with a second check by another review author. Study characteristics including design, patient population, and AMR metrics were extracted. We also extracted whether the authors indicated infection prevention and control (IPAC) measures were strengthened during the pandemic and/or whether there was an antimicrobial stewardship program (ASP) in place. This was categorized into two groups: (a) reporting of enhanced IPAC or ASP or (b) reported no enhanced IPAC/ASP OR did not report enhanced IPAC/ASP. These variables were extracted to stratify changes in AMR based on potential AMR-mitigating factors.

### Risk of bias assessment

We used a 10-item validated risk of bias in prevalence studies tool incorporated into data extraction [11].

### Data analysis

Findings were summarized descriptively. In studies providing complete numerator and denominator data, incidence rate ratio (IRR) were pooled using a GLMM random-effects meta-analysis and risk ratio (RR) were pooled using Mantel-Haenszel random-effects meta-analysis with between-study variance estimated using the Paule-Mandel estimator. Results were presented in forest plots and pooled across Gram-positive and Gram-negative organisms, stratified by the reporting of enhanced IPAC measures and/or ASP. All analyses were carried out using R version 4.1.2 with the packages *metafor* and *meta.*

Heterogeneity was assessed using the I^2^ statistic, with <40% considered low heterogeneity, 30%–60% considered moderate heterogeneity, 50%–90% considered substantial heterogeneity, and 75%–100% considered considerable heterogeneity [12]. Data and code are available at https://github.com/jeanpaulrsoucy/covid-19-amr-meta-analysis.

## Results

**Fig. 1:**
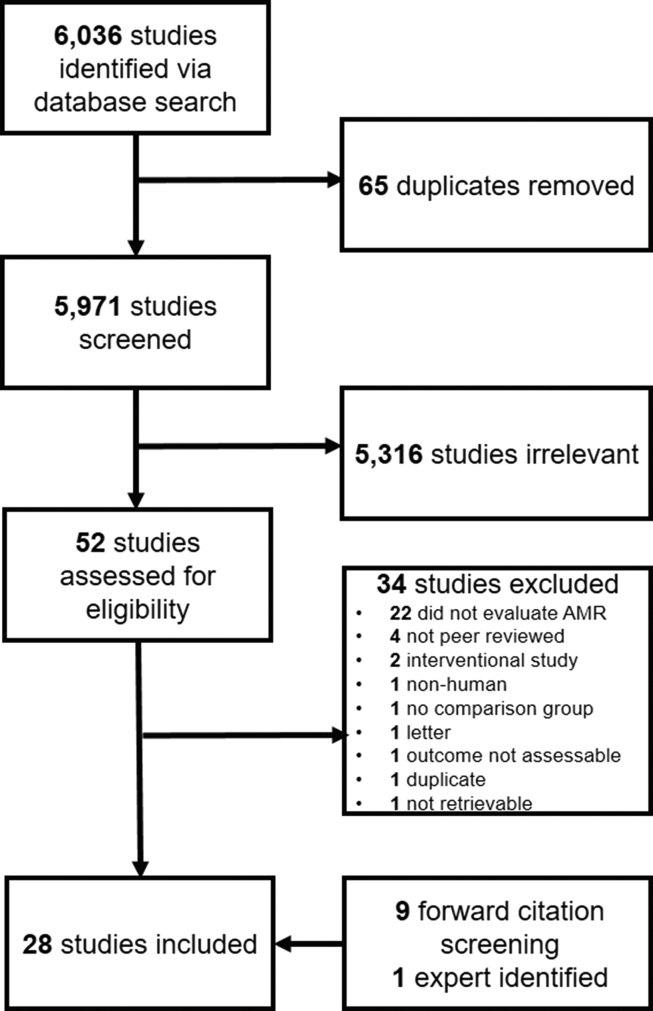
Preferred Reporting Items for Systematic Reviews and Meta-Analyses flow diagram.

Of 6036 studies identified via literature search, 28 were eligible for inclusion (18 via full-text screening, 9 via forward citation screening, and 1 expert-identified; Fig. 1) [[13], [14], [15], [16], [17], [18], [19], [20], [21], [22], [23], [24], [25], [26], [27], [28], [29], [30], [31], [32], [33], [34], [35], [36], [37], [38], [39], [40]]. The most common countries of origin were the United States (*n* = 4), Italy (*n* = 4), and Brazil (*n* = 3). Patient populations studied included all hospitalized patients (*n* = 17), those hospitalized in intensive care units only (*n* = 5), special populations (e.g. oncology, surgery) (*n* = 3), mixed hospitalized and community-dwelling patients (*n* = 2), and community-dwelling patients only (*n* = 1). Studies evaluated a range of both community- and health care–acquired infections. Combined health care and community-acquired infection or setting of acquisition commonly undistinguished (*n* = 15), followed by only health care-associated (*n* = 11), and only community-acquired (*n* = 2). The majority of studies derived resistance data from clinical specimens (*n* = 20), six included both clinical and screening specimens or did not specify the type of specimen, followed by two studies using screening specimens only. Most studies had a moderate risk of bias (*n* = 18), followed by low (*n* = 5), and high (*n* = 5) risk of bias (Table 1). The most common reasons for the downgraded risk of bias included inconsistent or lack of reporting on the mode of data collection across study subjects, lack of reporting of case definitions, and lack of reporting of complete numerator and denominator data (Table S1).

**Table 1:** Antimicrobial resistance associated with COVID-19 study characteristics

Author, year	Country	Health care setting	Acquisition	AMR measure	Directionality^a^						Risk of bias
					MRSA	VRE	PsA	Abau	ESBL	CRE	
Baker MA [13], 2021	United States	All hospitalized	Health care–acquired	Incidence per COVID-19 rate	↑	↑					Moderate
Belvisi V [14], 2021	Italy	ICU only	Not specified	Point prevalence, incidence density						↑	High
Bentivegna E [15], 2021	Italy	All hospitalized	Health care–acquired	Incidence	↓		↓				High
Castro MG [16], 2022	Argentina	All hospitalized	Both	Incidence density						↑	Moderate
Chamieh A [17], 2021	Lebanon	All hospitalized	Both	Incidence density		↓	↑	↓		↓	Moderate
Despotovic A [18], 2021	Serbia	ICU only	Health care–acquired	Proportion		↑	↑	↑	↓	↑	Moderate
Evans ME [19], 2022	United States	All hospitalized	Health care–acquired	Incidence density	↑						Moderate
Gaspar GG [20], 2021	Brazil	ICU only	Not specified	Incidence density				↑			Moderate
Gisselo KL [21], 2022	Denmark	All hospitalized	Health care–acquired	Incidence		↓					Low
Guven DC [22], 2021	Turkey	Palliative oncology	Health care–acquired	Proportion	↑				↑	↑	Moderate
Hirabayashi A [23], 2021	Japan	All hospitalized	Both	Proportion	↑		↑		↑		Moderate
Jeon [24]K, 2022	South Korea	ICU only	Both	Incidence density	↓	↑	↓	↓		↑	Moderate
La Vecchia A [25], 2022	Italy	All hospitalized	Both	Proportion	↓						Moderate
Lemenand O [26], 2021	France	Community	Community-acquired	Proportion					↓		Low
Lo SH [27], 2020	Taiwan	All hospitalized	Not specified	Incidence density	↓	↓	↑	↓			Moderate
Mares C [28], 2022	Romania	Hospitalized/non-hospitalized	Both	Proportion		↑	↑		↑	↑	Moderate
McNeil MJ [29], 2021	United States	All hospitalized	Community-acquired	Incidence	↓						Moderate
Micozzi A [30], 2021	Italy	Malignant haematology	Both	Proportion						↓	Low
O'Riordan F [31], 2022	Ireland	All hospitalized	Both	Proportion		↓			↓	↑	High
Ochoa-Hein E [32], 2021	Mexico	All hospitalized	Health care–acquired	Incidence density	↓		↑	↓	↑	↑	Moderate
Polemis M [33], 2021	Greece	All hospitalized	Both	Proportion	↑	↑	↓	↑		↑	Moderate
Polly M [34], 2022	Brazil	All hospitalized	Health care–acquired	Incidence density	↑	↓	↓	↑		↑	Moderate
Porto APM [35], 2022	Brazil	ICU only	Health care–acquired	Incidence density	↑	↑				↑	Low
Tham N [36], 2022	Australia	Surgical	Health care–acquired	Incidence	↑	↑			↑		Moderate
Tizkam HH [37], 2020	Iraq	All hospitalized	Not specified	Proportion			↑		↑	↑	High
Wardoyo EH [38], 2021	Indonesia	Hospitalized/non-hospitalized	Not specified	Proportion					↓		High
Wee LEI [39], 2021	Singapore	All hospitalized	Both	Incidence density	↓		↑			↓	Low
Weiner-Lastinger LM [40], 2022	United States	All hospitalized	Health care–acquired	Standardized infection ratio	↑						Moderate

Abau, multi-drug resistant or carbapenem-resistant *Acinetobacter baumannii*; AMR, antimicrobial resistance; CRE, carbapenem-resistant Enterobacterales; ESBL, extended-spectrum beta-lactamase–producing organism; ICU, intensive care unit; MRSA, methicillin-resistant *S. aureus*; PsA, multi-drug resistant or carbapenem-resistant *Pseudomonas aeruginosa*; VRE, vancomycin-resistant Enterococcus.

a Directionality refers to a numerical change in AMR during COVID-19 compared with before the pandemic.

### Measures of AMR

Incidence density (e.g. cases of resistant infections per 1000 patient days) was most commonly used to measure a change in AMR associated with COVID-19 (*n* = 11) or proportion of isolates or infections (e.g. percentage of *S. aureus* cases that were MRSA, *n* = 11), followed by incidence (e.g. cases per admission or discharges, *n* = 5), and other (standardized infection ratio, point prevalence *n* = 2). Study details and AMR metric directionality are provided in Table 1. Of the 28 eligible studies, 23 (82%) provided raw numerator and denominator data to facilitate meta-analysis.

### Resistance in Gram-positive organisms

#### Methicillin-resistant Staphylococcus aureus

Over 6,848,357 patient days of follow-up, our meta-analysis found that the COVID-19 pandemic was not associated with a change in the incidence rate of MRSA (IRR 1.03, 95% CI: 0.65–1.62, I^2^ = 95%, *n* = 5). Similarly, the COVID-19 pandemic was not associated with a change in the proportion of cases that were MRSA (RR 0.91, 95% CI: 0.60–1.36, I^2^ = 93%, *n* = 7).

### Vancomycin-resistant enterococci

Over 356,056 patient days, meta-analysis shows that the COVID-19 pandemic was not associated with a change in the incidence of VRE (IRR 0.75, 95% CI: 0.49–1.15, I^2^ = 56%, *n* = 3). Similarly, there was no change in the proportion of VRE cases (RR 0.91, 95% CI: 0.30–2.79, I^2^ = 94%, *n* = 5).

### Overall Gram-positive resistance and association with infection prevention and antimicrobial stewardship initiatives

**Fig. 2:**
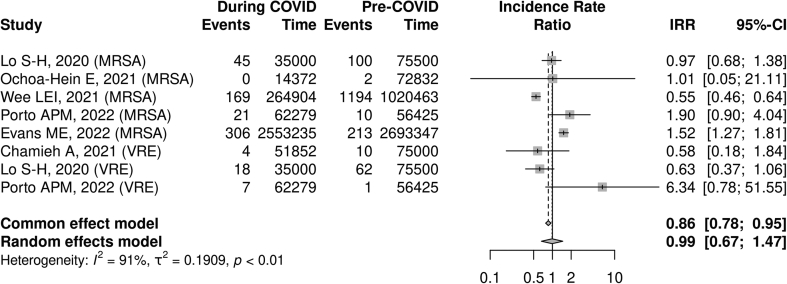
COVID-19 pandemic and Gram-positive antimicrobial resistance incidence rate ratio.^a^ ^a^All included studies reported infection prevention and control/antimicrobial stewardship program initiatives.

**Fig. 3:**
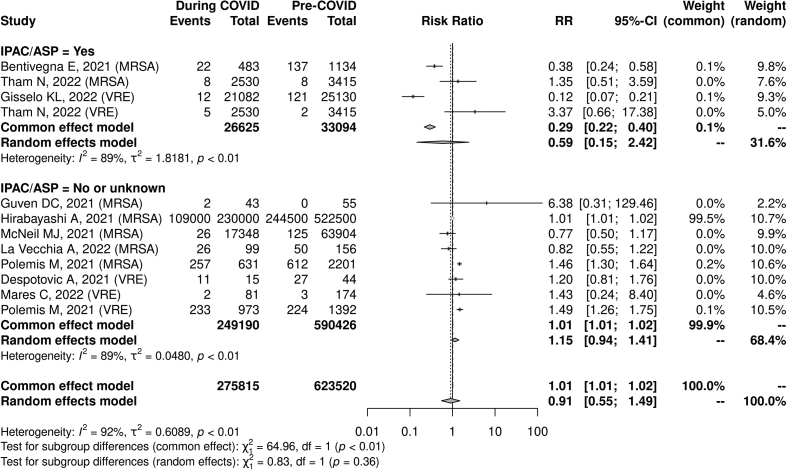
COVID-19 pandemic and Gram-positive antimicrobial resistance risk ratio and reported presence vs. absence of infection prevention and control/antimicrobial stewardship programs.^a^ ^a^Represents proportions of patients (e.g. visits) or organisms in which antimicrobial resistance was identified.

When pooling both MRSA and VRE, no association was found between the COVID-19 pandemic and the incidence (IRR 0.99, 95% CI: 0.67–1.47, I^2^ = 91%, *n* = 8) or proportion (RR 0.91, 95% CI: 0.55–1.49, I^2^ = 92%, *n* = 12) of resistant Gram-positive cases. The reported presence of IPAC or ASP interventions was not associated with a statistically significant difference in resistance rates (reporting IPAC/ASP: RR 0.59, 95% CI 0.15–2.42, I^2^ = 89%, *n* = 4; not reporting IPAC/ASP: RR: 1.15, 95% CI: 0.94–1.41, I^2^ = 89% *n* = 8, test of subgroup difference p 0.36; see Fig. 2, Fig. 3).

### Resistance in Gram-negative organisms

#### Resistant Acinetobacter baumannii

Across 325,847 patient days, there was no association between COVID-19 and the incidence of carbapenem- or multi-drug-resistant *Acinetobacter* spp. (IRR 0.79, 95% CI: 0.30–2.07, I^2^ = 77%, *n* = 4). However, there was a small increase in the proportion of infections that were resistant to *Acinetobacter* spp. (RR 1.02, 95% CI: 1.01–1.03, I^2^ = 0%, *n* = 2).

#### Resistant *Pseudomonas aeruginosa*

Across 1,609,923 patient days, there was no association between COVID-19 and the incidence of resistant *Pseudomonas* (IRR 1.10, 95% CI: 0.91–1.30, I^2^ = 0%, *n* = 4). Similarly, there was no association with the proportion of resistant cases (RR 1.02, 95% CI: 0.85–1.23, I^2^ = 58%, *n* = 6).

### ESBL-producing (or third-generation cephalosporin-resistant) Enterobacterales

One study with 87,204 patient days of follow-up found an increased IRR associated with the COVID-19 pandemic (IRR 15.20, 95% CI: 4.90–47.14). However, the proportion of cases with an ESBL-producing organism was not significantly altered with COVID-19 (RR: 1.10, 95% CI: 0.91–1.33, I^2^ = 94%, *n* = 8).

### Carbapenem-resistant Enterobacterales

Across 587,047 patient days, there was no significant change detected in the incidence of CRE (*E. coli* and *Klebsiella* spp.) (IRR 2.05, 95% CI: 0.77–5.44, I^2^ = 95%, *n* = 5). Similarly, there was no identified increase in the proportion of cases that were CRE (RR 1.10, 95% CI: 0.61–1.99, I^2^ = 88%, *n* = 6).

### Overall Gram-negative resistance and association with infection prevention and antimicrobial stewardship

**Fig. 4:**
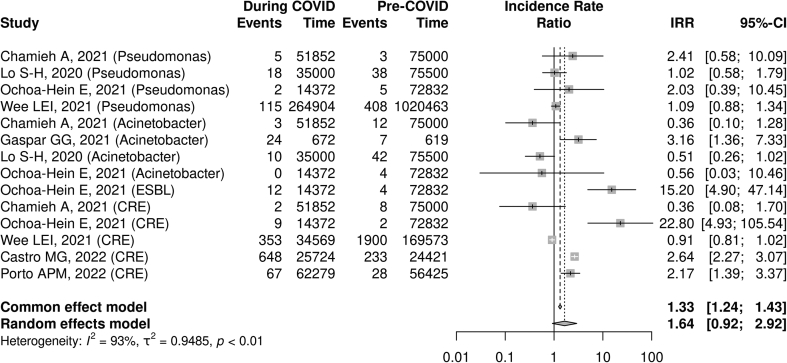
COVID-19 pandemic and Gram-negative antimicrobial resistance incidence rate ratio.^a^ ^a^All included studies reported infection prevention and control/ASP initiatives.

**Fig. 5:**
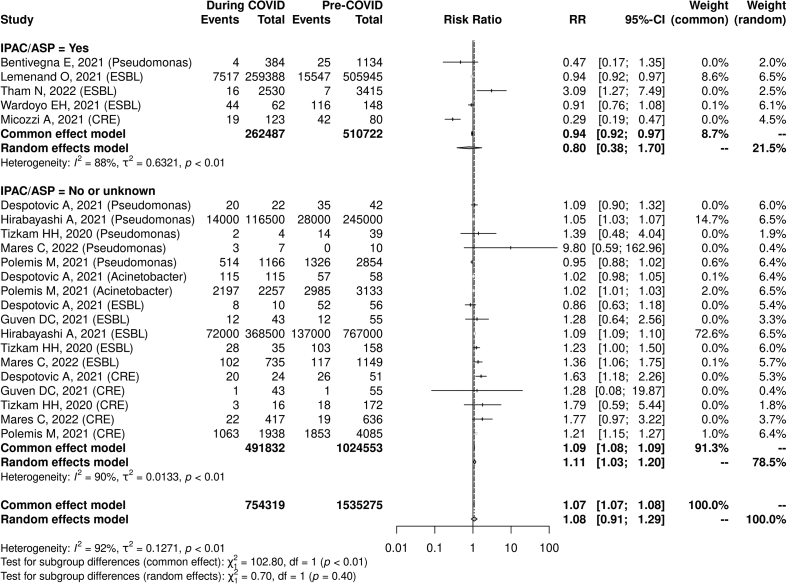
COVID-19 pandemic and Gram-negative antimicrobial resistance risk ratio and reported presence vs. absence of infection prevention and control/antimicrobial stewardship programs.∗ ∗Represents proportions of patients (e.g. visits) or organisms in which antimicrobial resistance was identified.

When pooling all resistant Gram-negative organisms, there was a non-statistically significant association between the COVID-19 pandemic and the incidence rate (IRR 1.64, 95% CI: 0.92–2.92, I^2^ = 93%, *n* = 14) as well as the proportion of cases that were resistant (RR 1.08, 95% CI: 0.91–1.29, I^2^ = 92%, *n* = 22). The lack of reporting of enhanced IPAC and/or ASP was significantly associated with an increase in gram-negative AMR (RR 1.11, 95% CI: 1.03–1.20, I^2^ = 88%, *n* = 5), whereas no significant association with AMR was seen in studies that did report such initiatives (RR 0.80, 95% CI: 0.38–1.70, I^2^ = 90%, *n* = 17). A test for subgroup differences showed no statistically significant difference between the presence and absence of reported enhanced IPAC/ASP interventions when evaluating changes in AMR (p = 0.40; Fig. 4, Fig. 5).

## Discussion

AMR definitions and reporting in the context of the COVID-19 pandemic are variable with substantial heterogeneity in reported outcomes among studies. We found that although the COVID-19 pandemic was not associated with a change in Gram-positive AMR, Gram-negative resistance may have increased (MDR or carbapenem-resistant *P. aeruginosa* or *Acinetobacter spp.*, ESBL and CRE), particularly in settings where enhanced IPAC and ASP initiatives were not reported.

A recent special report from the US Centers for Disease Control and Prevention found a 15% increase in the rate (per discharge or admission) of resistant organisms including carbapenem-resistant *Acinetobacter*, MRSA, CRE, and ESBL associated with the pandemic [8]. Study location, the burden of COVID-19, the burden of non-COVID-19 respiratory infections, background epidemiology, and antimicrobial prescribing practices may partially explain the difference between the Centers for Disease Control and Prevention data and our findings. The apparent increase in the incidence of Gram-negative AMR but not Gram-positive AMR suggests antibiotic prescribing may play an important role, given the high use of beta-lactam/beta-lactamase inhibitors and third-generation cephalosporins in patients with COVID-19 [3]. Nevertheless, both the Centers for Disease Control and Prevention report and this systematic review present a concern that the COVID-19 pandemic may play a role in increasing rates of AMR in the population.

The concern for increasing AMR in the context of COVID-19 has been previously highlighted [41,42]. Inappropriately high usage of antibiotics in patients with COVID-19 selects for resistant organisms which can potentially be transmitted to the broader population [42]. We have previously shown that in the context of COVID-19 co-infection and secondary infections, 38% of organisms and 61% of patients harbour AMR [4]. This analysis extends the concern for drug resistance beyond patients with COVID-19 themselves to document an ecologic impact of the pandemic on AMR.

The relationship between COVID-19 and AMR is complex, as several factors such as improved hand hygiene, personal protective equipment use, and physical distancing may help to reduce the transmission of AMR organisms, at least temporarily while such enhanced measures are in place [7]. On the other hand, a shortage of medical personnel and personal protective equipment during the pandemic could thwart this effect. Our findings reinforce that IPAC activities are important mitigating factors limiting the growth of AMR associated with COVID-19.

Although this study provides a broad global view of AMR in the context of COVID-19 from an ecological perspective, there are several important limitations. There is significant heterogeneity in methodology and AMR outcome measures reported across studies. At least five different AMR metrics were provided (incidence density, incidence per admission/discharge, proportion of infections, standardized infection ratio, and point prevalence), which prevents direct comparison and makes meta-analysis challenging. A lack of adjustment for confounding factors raises the possibility that changes in AMR over time may be due to changes in patient populations or other underlying factors. And lack of longitudinal data also limits the ability to account for existing temporal trends in AMR incidence and prevalence, as well as more distal changes that continue to evolve after the pandemic. Differences in regional baseline rates of AMR or epidemiology may also account for the heterogeneity seen, and as such individualized assessment of regional AMR surveillance data are needed. Many studies did not comment on other confounding factors such as the presence or intensity of their IPAC or ASP, so in studies reporting these interventions, the association with reduced AMR may represent correlation rather than causation. Several studies only reported a small number of AMR phenotypes, hence there is a risk of selective outcome reporting. It is also important to note that less than 20% of studies had a low risk of bias, suggesting that higher quality studies are needed to better understand the impact of COVID-19 on AMR.

## Conclusion

The COVID-19 pandemic could play an important role in the emergence and transmission of resistant pathogens, particularly for Gram-negative organisms in hospital settings. There is considerable heterogeneity in both the AMR metrics used and the rate of resistance reported across studies. Our findings reinforce not only the need for strengthened infection prevention and antimicrobial stewardship but also robust and consistent AMR surveillance as part of the pandemic response and recovery.

## Author contributions

All authors conceptualized and designed the study and performed acquisition, analysis, or interpretation of data. BJL drafted the manuscript. All authors critically revised the manuscript for important intellectual content. J-PRS and BJL performed statistical analysis. All authors provided administrative, technical, or material support.

## Transparency declaration

The authors declare that they have no conflicts of interest. SB is the staff at WHO. This paper solely reflects the view of the authors and does not necessarily reflect the view of the organization. This study was supported by funding from the 10.13039/100004423WHO. This work was previously published as a pre-print: https://doi.org/10.1101/2022.09.01.22279488.
