# Supplementary material for: Antibiotic resistance associated with the COVID-19 pandemic: a systematic review and meta-analysis
Source: Clin Microbiol Infect. 2023 Mar;29(3):302–9. doi: 10.1016/j.cmi.2022.12.006 (PMC9733301; doi:10.1016/j.cmi.2022.12.006)
Supplement: Supplementary material [file mmc1.docx]

Supplementary Table 1. Risk of Bias for Included Studies

| Author, Year | Target population representativeness? | Sampling Frame representative ness? | Random selection? | Minimal non-response? | Data collection directly from subjects? | Acceptable case definition? | Valid and reliable study instrument? | Consistent mode of data collection? | Appropriate duration of assessment? | Appropriate numerator and denominator | Quality Score |
| --- | --- | --- | --- | --- | --- | --- | --- | --- | --- | --- | --- |
| Baker MA, 2021 | Yes | No or unclear | Yes | Yes | Yes | Yes | No or unclear | No or unclear | Yes | Yes | 7 |
| Belvisi V, 2021 | No or unclear | Yes | No or unclear | Yes | Yes | No or unclear | No or unclear | No or unclear | Yes | No or unclear | 4 |
| Bentivegna E, 2021 | No or unclear | Yes | No or unclear | Yes | Yes | No or unclear | No or unclear | No or unclear | No or unclear | Yes | 4 |
| Castro MG, 2022 | No or unclear | Yes | Yes | Yes | Yes | No or unclear | Yes | No or unclear | Yes | No or unclear | 6 |
| Chamieh A, 2021 | No or unclear | No or unclear | Yes | Yes | Yes | No or unclear | Yes | No or unclear | Yes | No or unclear | 5 |
| Despotovic A, 2021 | No or unclear | Yes | Yes | Yes | Yes | Yes | No or unclear | No or unclear | No or unclear | No or unclear | 5 |
| Evans ME, 2022 | No or unclear | Yes | Yes | Yes | Yes | Yes | No or unclear | No or unclear | Yes | Yes | 7 |
| Gaspar GG, 2021 | No or unclear | Yes | No or unclear | Yes | Yes | No or unclear | Yes | No or unclear | Yes | No or unclear | 5 |
| Gisselo KL, 2022 | No or unclear | Yes | Yes | Yes | Yes | Yes | Yes | No or unclear | Yes | Yes | 8 |
| Guven DC, 2021 | Yes | Yes | Yes | Yes | Yes | Yes | No or unclear | No or unclear | No or unclear | No or unclear | 6 |
| Hirabayashi A, 2021 | Yes | Yes | Yes | Yes | Yes | No or unclear | Yes | No or unclear | Yes | No or unclear | 7 |
| Jeon K, 2022 | No or unclear | Yes | Yes | Yes | Yes | No or unclear | Yes | No or unclear | Yes | No or unclear | 6 |
| La Vecchia A, 2022 | No or unclear | Yes | No or unclear | Yes | Yes | Yes | Yes | No or unclear | Yes | No or unclear | 6 |
| Lemenand O, 2021 | Yes | Yes | Yes | Yes | Yes | Yes | No or unclear | No or unclear | Yes | Yes | 8 |
| Lo S-H, 2020 | No or unclear | Yes | Yes | Yes | Yes | No or unclear | No or unclear | No or unclear | Yes | No or unclear | 5 |
| Mares C, 2022 | No or unclear | Yes | Yes | Yes | Yes | No or unclear | Yes | No or unclear | Yes | Yes | 7 |
| McNeil MJ, 2021 | No or unclear | Yes | Yes | Yes | Yes | No or unclear | Yes | No or unclear | Yes | No or unclear | 6 |
| Micozzi A, 2021 | Yes | Yes | No or unclear | Yes | Yes | No or unclear | Yes | Yes | Yes | Yes | 8 |
| O'Riordan F, 2022 | No or unclear | Yes | No or unclear | Yes | Yes | No or unclear | No or unclear | No or unclear | Yes | No or unclear | 4 |
| Ochoa-Hein E, 2021 | No or unclear | Yes | Yes | Yes | Yes | Yes | No or unclear | No or unclear | Yes | No or unclear | 6 |
| Polemis M, 2021 | No or unclear | Yes | No or unclear | Yes | Yes | No or unclear | Yes | No or unclear | Yes | Yes | 6 |
| Polly M, 2022 | No or unclear | Yes | Yes | Yes | Yes | Yes | No or unclear | No or unclear | Yes | Yes | 7 |
| Porto APM, 2022 | Yes | Yes | Yes | Yes | Yes | Yes | Yes | No or unclear | Yes | No or unclear | 8 |
| Tham N, 2022 | Yes | Yes | Yes | Yes | Yes | No or unclear | No or unclear | No or unclear | Yes | Yes | 7 |
| Tizkam HH, 2020 | No or unclear | No or unclear | No or unclear | No or unclear | Yes | No or unclear | Yes | No or unclear | Yes | No or unclear | 3 |
| Wee LEI, 2021 | No or unclear | Yes | Yes | Yes | Yes | Yes | Yes | Yes | Yes | Yes | 9 |
| Wardoyo EH, 2021 | No or unclear | No or unclear | No or unclear | Yes | Yes | No or unclear | Yes | No or unclear | Yes | Yes | 5 |
| Weiner-Lastinger LM, 2022 | No or unclear | Yes | Yes | Yes | Yes | Yes | No or unclear | No or unclear | Yes | Yes | 7 |

Full list of risk of bias questions available at: Hoy D, Brooks P, Woolf A, Blyth F, March L, Bain C, Baker P, Smith E, Buchbinder R. Assessing risk of bias in prevalence studies: modification of an existing tool and evidence of interrater agreement. J Clin Epidemiol. 2012 Sep;65(9):934-9. doi: 10.1016/j.jclinepi.2011.11.014. Epub 2012 Jun 27. PMID: 22742910.
